# Folate-Targeted Nanocarriers Co-Deliver Ganciclovir and miR-34a-5p for Combined Anti-KSHV Therapy

**DOI:** 10.3390/ijms25052932

**Published:** 2024-03-02

**Authors:** Fangling Li, Dongdong Cao, Wenyi Gu, Dongmei Li, Zhiyong Liu, Lin Cui

**Affiliations:** 1 Key Laboratory of Xinjiang Endemic and Ethnic Diseases, NHC Key Laboratory of Prevention and Treatment of Central Asia High Incidence Diseases, School of Medicine, Shihezi University, Shihezi 832002, China; lifanglingcdd@sina.com (F.L.); caodongd@shzu.edu.cn (D.C.); cuilin@shzu.edu.cn (L.C.); 2 State Key Laboratory Incubation Base for Green Processing of Chemical Engineering, School of Chemistry and Chemical Engineering, Shihezi University, Shihezi 832003, China; 3 Australian Institute for Bioengineering and Nanotechnology (AIBN), University of Queensland (UQ), Corner College and Cooper Roads (Building 75), Brisbane, QLD 4072, Australia; w.gu@uq.edu.au

**Keywords:** anti-KSHV therapy, ganciclovir, miR-34a-5p, co-loaded dual-drug nanocomplex, combination of two drugs

## Abstract

Kaposi’s sarcoma-associated herpesvirus (KSHV) can cause a variety of malignancies. Ganciclovir (GCV) is one of the most efficient drugs against KSHV, but its non-specificity can cause other side effects in patients. Nucleic acid miR-34a-5p can inhibit the transcription of KSHV RNA and has great potential in anti-KSHV therapy, but there are still problems such as easy degradation and low delivery efficiency. Here, we constructed a co-loaded dual-drug nanocomplex (GCV@ZIF-8/PEI-FA+miR-34a-5p) that contains *GCV* internally and adsorbs miR-34a-5p externally. The folic acid (FA)-coupled polyethyleneimine (PEI) coating layer (PEI-FA) was shown to increase the cellular uptake of the nanocomplex, which is conducive to the enrichment of drugs at the KSHV infection site. *GCV* and miR-34a-5p are released at the site of the KSHV infection through the acid hydrolysis characteristics of ZIF-8 and the “proton sponge effect” of PEI. The co-loaded dual-drug nanocomplex not only inhibits the proliferation and migration of KSHV-positive cells but also decreases the mRNA expression level of KSHV lytic and latent genes. In conclusion, this co-loaded dual-drug nanocomplex may provide an attractive strategy for antiviral drug delivery and anti-KSHV therapy.

## 1. Introduction

Kaposi’s sarcoma-associated herpesvirus (KSHV) belongs to the gamma-herpesvirus subfamily and is closely related to malignant tumors [1,2,3]. It is a significant threat to the life and health of human beings, especially in patients with low immune function [4]. At present, the main treatment methods for KSHV-related malignancies are anti-KSHV drugs that block viral DNA replication [5,6,7].

Ganciclovir (GCV), also named 9-(1,3-dihydroxy-2-propoxymethyl) guanine, is a white crystalline powder that is a cyclic analog of endogenous nucleoside guanine and a small-molecule antiviral drug that inhibits viral genome replication by inhibiting the DNA polymerase protein of KSHV [8,9,10]. However, the transmembrane capacity of *GCV* is poor, and long-term intravenous administration is required to achieve the required concentration for treatment, which leads to various types of thrombocytopenia and persistent myelosuppression [11]. miR-34a-5p is a small molecule–nucleic acid belonging to the miR-34 family and has been proven to play an important role in the treatment of KSHV [12]. However, like other nucleic acid drugs, miR-34a-5p exhibits a low cell uptake efficiency and rapid enzymatic hydrolysis in vivo, and it cannot exert the expected therapeutic effect [13,14], which limits its clinical application.

With the rapid development of nanomaterials and medical technology, nanomedicine delivery systems have been developed to solve many problems associated with free drugs in the treatment of tumors [15,16,17]. In terms of anti-KSHV therapy, our team previously developed a nano delivery vector based on cationic polymer polyethylenimide for the targeted delivery of miR-34a-5p, while improving its instability [18]. Due to the complexity of the KSHV infection site, monotherapeutic agents cannot achieve satisfactory therapeutic effects. At present, combination therapy based on nanotechnology is becoming an important model of the advanced enhancement of anti-cancer effects [19,20,21,22]. One promising anti-KSHV strategy is the design of nanocarriers that can simultaneously load *GCV* and miR-34a-5p and deliver these two drugs to the KSHV infection site as an anti-KSHV drug combination. The preparation of such nanocarriers capable of delivering both antiviral drugs and nucleic acids remains challenging based on the need to ensure that the two drugs do not interfere with each other during administration and that the drug’s anti-KSHV benefits are maximized.

Zeolite imidazole framework-8 (ZIF-8) is a class of metal–organic framework (MOF) materials with a zeolite skeleton structure. Compared with traditional drug delivery systems, ZIF-8 has the advantages of adjustable pores, a large specific surface area, a strong drug loading ability, a simple synthesis process, and responsive drug loading under mild and slightly acidic conditions [23,24]. Importantly, ZIF-8 is currently being extensively studied as a nanocarrier and has been shown to support various types of drugs such as chemotherapy drugs [25,26], genes [27,28], nucleic acid drugs [29,30], proteins [31], antibiotics [32], and photothermal agents [33,34]. Inspired by natural biological activation processes, Liu et al. [35] constructed a biomimetic nanoplatform for the co-delivery of the nucleic acid drug G3139 (an antisense oligonucleotide capable of targeting the anti-apoptosis protein Bcl-2) and the chemotherapy drug doxorubicin (DOX). G3139, DOX, and Fe^2+^ ions can have coordination interactions, and with the help of Fe^2+^ ions, these two types of drugs assemble together into uniform particles (DOX/Fe-G). The resulting DOX/Fe-G is then mineralized with ZIF-8 in thin shells to produce the final core–shell nanoplatform DOX/Fe-G@Z. The results showed that the nanoplatform enhanced the anti-cancer capabilities and allowed for in vivo magnetic resonance imaging. This method of simultaneously encapsulating a chemotherapy drug and a nucleic acid drug through the “one-pot method” is a very simple approach, but it also requires the two drugs to be encapsulated to have good stability.

Many studies have reported that positively charged materials can be combined with negatively charged genes and nucleic acids through electrostatic adsorption to form drug-carrying nanocomplexes for the effective delivery of genes and nucleic acids [36]. Wang et al. [37] prepared a multifunctional nanoparticle based on quantum dots (CDs) and modified β-cyclodextrin (β-CD) and arginine-glycine–aspartate (RGD) peptides. The nanocarrier can not only carry the small-molecule chemotherapeutic drug 5-fluorouracil (5-FU) through the hydrophobic cavity of β-CD, but also adsorb the electronegative siRNA (miR-10b inhibitor) through the positive electronegativity of the RGD peptide. In addition, RGD peptide also has the targeting properties of epithelial growth factor receptor (EGFR), which can deliver chemotherapy and nucleotide drugs to different targets of colorectal cancer, effectively inhibiting tumor growth and metastasis. This strategy of loading a chemotherapeutic drug and siRNA into different positions on a nanocarrier at the same time can sufficiently prevent the two drugs from interfering with each other, but the stability of the drug is not very high, and the responsive release of the drug at the tumor site is still lacking. These results indicate that when a nanocarrier is loaded with two drugs at the same time, it is necessary to design the drug loading mode and means of release according to the properties of the two drugs, which is very important for the subsequent drug combination therapy in cancer.

**Scheme 1:**
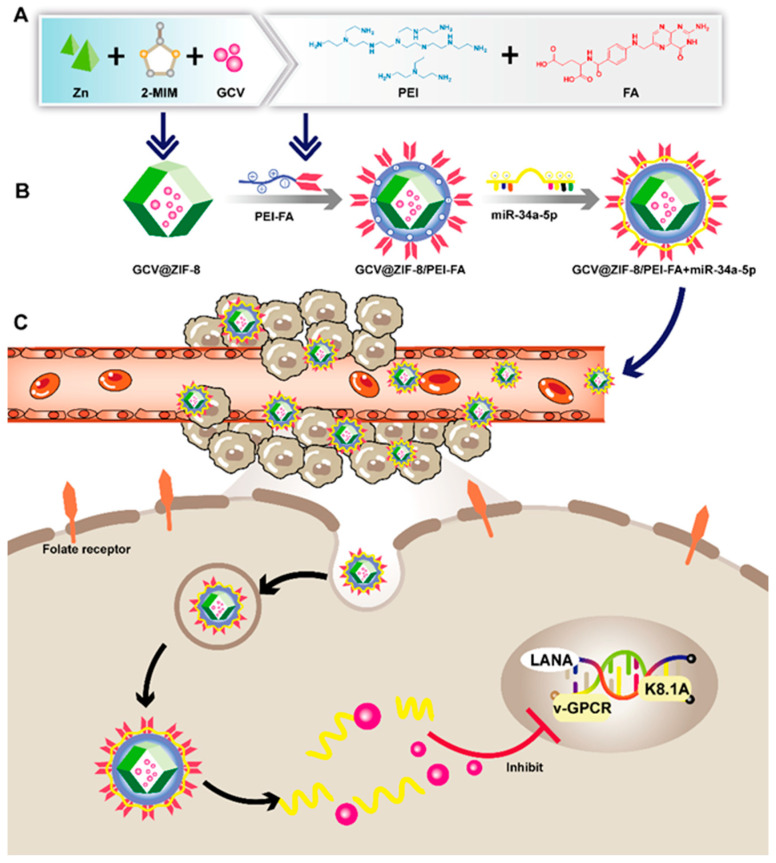
(**A**) GCV@ZIF-8 and PEI-FA synthesis route, (**B**) GCV@ZIF-8/PEI-FA+miR-34a-5p synthesis route, and (**C**) folate-receptor-mediated intracellular delivery of *GCV* and miR-34a-5p for combined anti-KSHV schematic.

In this study, a nano-drug delivery system was constructed based on the principle of layer-by-layer coating to solve the problems of non-specificity, toxicity and side effects, easy degradation, and the low uptake rate of cells that exist in the direct administration of *GCV* and miR-34a-5p. First, *GCV* was loaded into ZIF-8 using a “one-pot method” to form the drug-carrying nanoparticles GCV@ZIF-8. Then, folic acid (FA)-coupled PEI was coated on the outer surface of the GCV@ZIF-8 to form the FA-targeting supported single-drug nanocarrier GCV@ZIF-8/PEI-FA. Finally, the GCV@ZIF-8/PEI-FA formed the co-loaded dual-drug nanocomplex GCV@ZIF-8/PEI-FA+miR-34a-5p through the electrostatic adsorption of miR-34a-5p (Scheme 1). An in vitro drug release experiment showed that *GCV* exhibited pH-response release behavior. Cell experiments demonstrated that the presence of the PEI-FA coating can increase the uptake of the co-loaded dual-drug nanocomplex through KSHV-infected cells. In addition, the co-loaded dual-drug nanocomplex can exert an anti-KSHV effect by inhibiting cell proliferation and migration, as well as decreasing the expression of mRNA in KSHV latent and lytic genes, and the dual-drug anti-KSHV effect was superior to that of single-drug *GCV* or miR-34a-5p. This study provides new possibilities for the treatment of KSHV in humans.

## 2. Results and Discussion

### 2.1. Preparation and Characterization of PEI-FA

**Figure 1:**
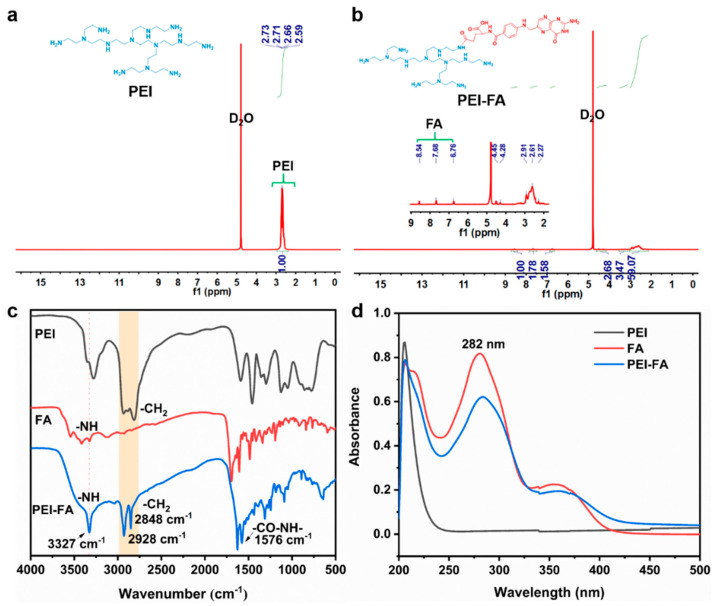
Structural characterization of PEI-FA: (**a**,**b**) Nuclear magnetic hydrogen spectra of PEI and PEI-FA, (**c**) infrared absorption spectra of PEI-FA, and (**d**) UV–Vis spectra of PEI-FA.

PEI-FA was synthesized according to Scheme 1. The carboxyl group on the FA was activated using DCC and NHS, and then grafted to PEI through an amide bond to form PEI-FA. Comparing the ^1^H NMR diagram of the raw material PEI (Figure 1a) with that of PEI-FA, we can see that the characteristic peak of hydrogen in the benzene ring of the FA appeared at 8.54–6.76 ppm, and the characteristic peak at 4.45–2.27 ppm was composed of PEI and FA together (Figure 1b). This indicates that the FA was successfully grafted onto the PEI. In order to further illustrate the results, the infrared and UV–Vis spectra of PEI-FA were tested. Figure 1c shows that 3327 cm^−1^ is the -NH bond-stretching vibration peak on the FA, 2928 cm^−1^ and 2848 cm^−1^ are the -CH_2_-CH_2_- stretching vibration peaks in PEI, and 1576 cm^−1^ is the -CO-NH- bond-stretching vibration peak. The absorption spectrum of PEI-FA is mainly composed of these peaks. Figure 1d shows that after grafting FA onto PEI, a characteristic peak of FA appeared at around 282 nm. All of the above results indicated that PEI-FA was successfully synthesized.

### 2.2. Preparation and Characterization of ZIF-8/PEI-FA and GCV@ZIF-8/PEI-FA

**Figure 2:**
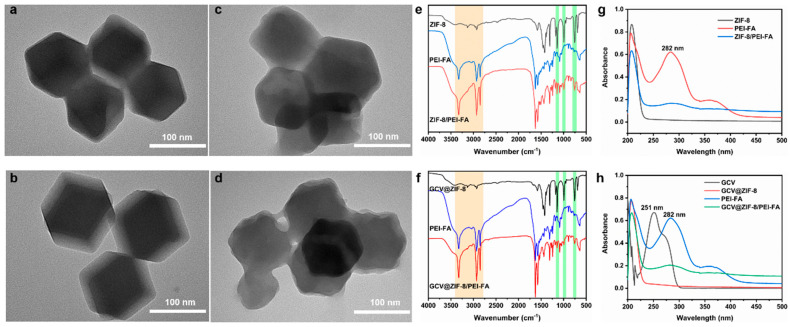
Morphologies and structural characterizations of ZIF-8/PEI-FA and GCV@ZIF-8/PEI-FA: TEM image of ZIF-8 (**a**), GCV@ZIF-8 (**b**), ZIF-8/PEI-FA (**c**), and GCV@ZIF-8/PEI-FA (**d**), (**e**,**g**) FTIR and UV–Vis spectra of ZIF-8/PEI-FA, and (**f**,**h**) FTIR and UV–Vis spectra of GCV@ZIF-8/PEI-FA.

TEM was used to verify whether the PEI-FA was successfully coated on the outer surfaces of ZIF-8 and GCV@ZIF-8. It can be seen from Figure 2a,b that the morphology of ZIF-8 is a dodecahedron. After the in situ encapsulation of GCV, the morphology of GCV@ZIF-8 was still a dodecahedron without significant changes, indicating that the encapsulation of *GCV* does not affect the morphology of ZIF-8. However, after coating with PEI-FA, the surfaces of ZIF-8 (Figure 2c) and GCV@ZIF-8 (Figure 2d) were covered by a layer of film and the edges became smooth, which indicates that ZIF-8 and GCV@ZIF-FA were successfully coated with PEI-FA. From Figure 2e,f, we can see that the infrared spectrum of GCV@ZIF-8 is almost identical to that of ZIF-8, indicating that *GCV* was indeed enclosed in the interior of ZIF-8. The infrared spectrum of ZIF-8/PEI-FA is mainly composed of the characteristic absorption peaks of ZIF-8 and PEI-FA, and the infrared spectrum of GCV@ZIF-8/PEI-FA is mainly composed of the characteristic absorption peaks of GCV@ZIF-8 and PEI-FA. In addition, the UV–Vis spectra shown in Figure 2g,h show that ZIF-8/PEI-FA and GCV@ZIF-8/PEI-FA exhibit characteristic absorption peaks of FA at around 282 nm, which also indicates that ZIF-8 and GCV@ZIF-FA were indeed coated with PEI-FA. All of the above results indicate that the ZIF-8/PEI-FA and GCV@ZIF-8/PEI-FA were successfully prepared.

**Figure 3:**
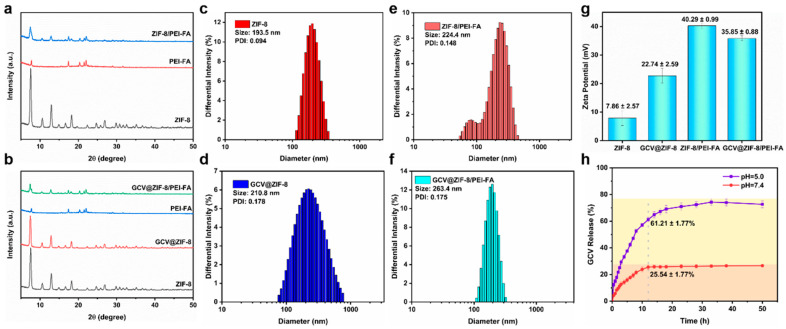
Structural characterizations of ZIF-8/PEI-FA and GCV@ZIF-8/PEI-FA: (**a**,**b**) XRD pattern of ZIF-8/PEI-FA and GCV@ZIF-8/PEI-FA, (**c**–**f**) particle size distribution diagram, (**g**) Zeta potential diagram, and (**h**) GCV@ZIF-8/PEI-FA cumulative release rate of *GCV* in *PBS* solution with pH = 7.4 and pH = 5.0 (*n* = 3, mean ± standard deviation, SD).

Next, XRD tests were conducted on the ZIF-8/PEI-FA and GCV@ZIF-8/PEI-FA. As shown in Figure 3a, the crystal diffraction peaks of ZIF-8/PEI-FA are mainly composed of the diffraction peaks of ZIF-8 and PEI-FA. The crystal diffraction peaks of GCV@ZIF-8/PEI-FA are mainly composed of GCV@ZIF-8 and PEI-FA, and the crystal diffraction peaks of GCV@ZIF-8 are basically consistent with those of ZIF-8 (Figure 3b). In addition, after coating with PEI-FA, the particle size and Zeta potential of the ZIF-8 and GCV@ZIF-8 changed. As shown in Figure 3c,e, the particle size of ZIF-8 increased from 193.5 nm to 224.4 nm, and that of GCV@ZIF-8 increased from 210.8 nm to 263.4 nm (Figure 3d,f). The potential of ZIF-8/PEI-FA was 40.29 ± 0.99 mV, and that of GCV@ZIF-8/PEI-FA was 35.85 ± 0.88 mV (Figure 3g). These changes also indicate that PEI-FA was successfully coated on the outer surfaces of the ZIF-8 and GCV@ZIF-8, and its positive potential also provides favorable conditions for the adsorption of miR-34a-5p.

### 2.3. Drug Loading Rate and In Vitro *GCV* Release Ability of GCV@ZIF-8/PEI-FA

In neutral conditions, ZIF-8 maintains structural stability, while under slightly acidic conditions, ZIF-8 decomposes to release drugs. This pH response characteristic makes ZIF-8 widely studied as a drug delivery carrier. Therefore, we also want to use this characteristic responsiveness to release GCV. Firstly, according to the standard curve (Figure S1) and Equation (1), the drug loading rate of the GCV@ZIF-8/PEI-FA was 8.6%. Then, the normal physiological environment and the slightly acidic environment of the tumor were simulated in vitro, and the cumulative drug release rate (Equation (2)) of *GCV* was calculated and plotted as a curve. The results are shown in Figure 3h. In the *PBS* solution with a pH of 7.4, the cumulative release rates of *GCV* at 12 h and 50 h were 25.54 ± 1.77% and 26.64 ± 0.86%, respectively. In *PBS* solution with pH 5.0, the cumulative *GCV* release rates at 12 h and 50 h were 61.21 ± 1.77% and 72.65 ± 2.59%, respectively. It can be concluded that GCV@ZIF-8/PEI-FA has certain stability under physiological conditions and can decompose and release *GCV* under slightly acidic tumor conditions. It can be used as a pH response drug delivery carrier for *GCV* to be released at the tumor site.

### 2.4. Preparation and Characterization of ZIF-8/PEI-FA+miR-34a-5p and GCV@ZIF-8/PEI-FA+miR-34a-5p

**Figure 4:**
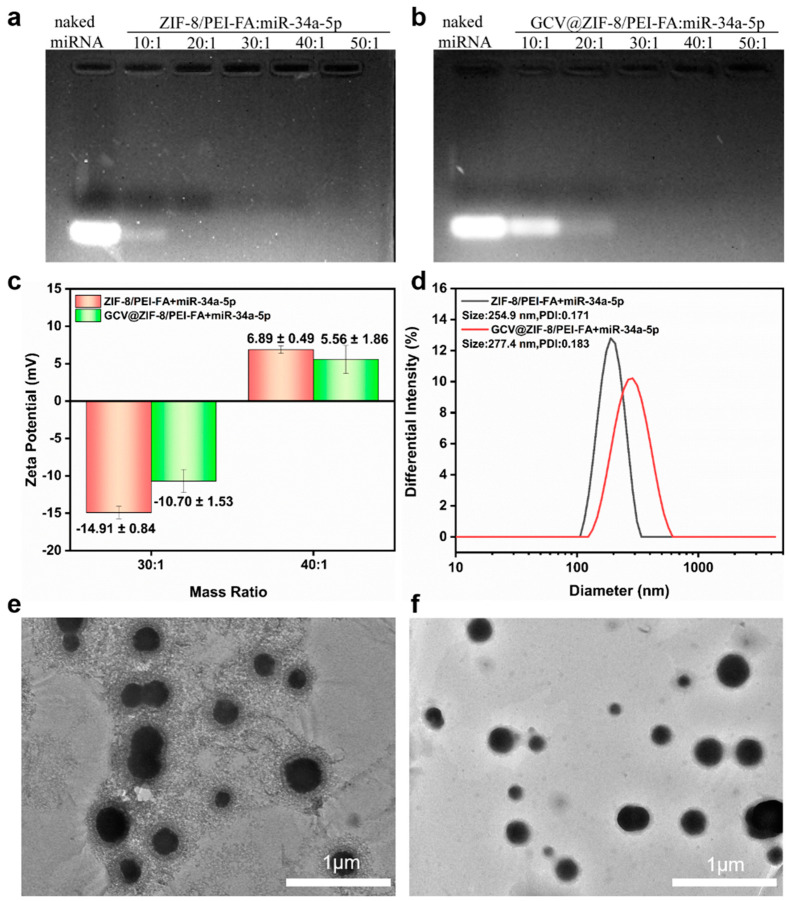
Structural characterizations of ZIF-8/PEI-FA+miR-34a-5p and GCV@ZIF-8/PEI-FA+miR-34a-5p: (**a**,**b**) gel electrophoresis images of ZIF-8/PEI-FA, GCV@ZIF-8/PEI-FA, and miR-34a-5p at different mass ratios, (**c**) Zeta potential images, (**d**) particle size distribution images, and (**e**,**f**) TEM images of ZIF-8/PEI-FA+miR-34a-5p and GCV@ZIF-8/PEI-FA+miR-34a-5p (scale bar: 1 μm). (*n* = 3, mean ± SD).

The supported single-drug nanocomplex ZIF-8/PEI-FA+miR-34a-5p and co-loaded dual-drug nanocomplex GCV@ZIF-8/PEI-FA+miR-34a-5p were prepared through the electrostatic absorption of the PEI-FA coating layers binding to miR-34a-5p (Scheme 1B). ZIF-8/PEI-FA, GCV@ZIF-8/PEI-FA, and miR-34a-5p were subjected to gel electrophoresis retardation experiments at different mass ratios to verify their binding ability. As shown in Figure 4a, when the mass ratio between ZIF-8/PEI-FA and miR-34a-5p was ≥20:1, miR-34a-5p was retarded in the upper sample hole by ZIF-8/PEI-FA, and no bright white bands appeared. As shown in Figure 4b, when the mass ratio was ≥30:1, miR-34a-5p was retarded in the upper sample hole by GCV@ZIF-8/PEI-FA, indicating that both substances can effectively bind and contain miR-34a-5p.

Subsequently, we detected the potential and particle size of ZIF-8/PEI-FA+miR-34a-5p and GCV@ZIF-8/PEI-FA+miR-34a-5p. As the mass ratio increased from 30:1 to 40:1, the potentials of ZIF-8/PEI-FA+miR-34a-5p and GCV@ZIF-8/PEI-FA+miR-34a-5p increased from negative to positive (Figure 4c), indicating that 40:1 is the optimal mass ratio for binding with miR-34a-5p. Under the condition of an optimal mass ratio, the particle sizes of ZIF-8/PEI-FA+miR-34a-5p and GCV@ZIF-8/PEI-FA+miR-34a-5p were 254.9 nm and 277.4 nm, respectively (Figure 4d), and their shapes were round (Figure 4e,f), which can be easily taken up by cells. All of the above results indicate that the coating layer of PEI-FA could play a role in the adsorption of nucleic acid drugs, and the carrier ZIF-8/PEI-FA could simultaneously support small-molecule antiviral drug *GCV* and miR-34a-5p.

### 2.5. Protective Effects of ZIF-8/PEI-FA and GCV@ZIF-8/PEI-FA on miR-34a-5p

**Figure 5:**
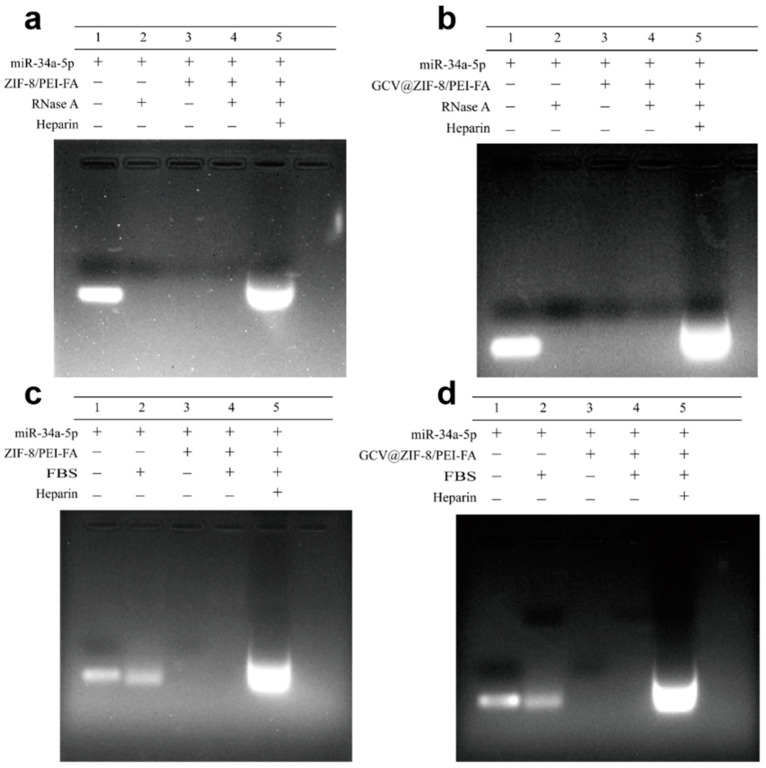
Protective effects of ZIF-8/PEI-FA and GCV@ZIF-8/PEI-FA on miR-34a-5p: (**a**,**b**) 0.05 mg/mL RNase A for 1 h and (**c**,**d**) 50% FBS for 1 h.

Since free miR-34a-5p is very unstable and easily decomposed, gel electrophoresis experiments were used to detect the protective effects of ZIF-8/PEI-FA and GCV@ZIF-8/PEI-FA on miR-34a-5p. As shown in Figure 5a,b, we can see bright white bands clearly in the free miR-34a-5p lane; when it co-cultured with RNase A, the white bands disappear, indicating that RNase A completely decomposed free miR-34a-5p. However, after ZIF-8/PEI-FA+miR-34a-5p and GCV@ZIF-8/PEI-FA+miR-34a-5p co-cultured with RNase A, no bands appeared. After the addition of heparin, which can compete with miR-34a-5p, the bands appeared again. These results indicate that ZIF-8/PEI-FA and GCV@ZIF-8/PEI-FA can protect miR-34a-5p by blocking it in the filling pore and preventing the decomposition of RNase A. Figure 5c,d show basically the same results: ZIF-8/PEI-FA and GCV@ZIF-8/PEI-FA can protect miR-34a-5p by preventing the decomposition of FBS. These results all indicate that ZIF-8/PEI-FA and GCV@ZIF-8/PEI-FA can effectively protect miR-34a-5p from decomposition through RNase A and decomposition in FBS, and are expected to improve the recycling of miR-34a-5p in vivo.

### 2.6. In Vitro Biosafety Analysis

**Figure 6:**
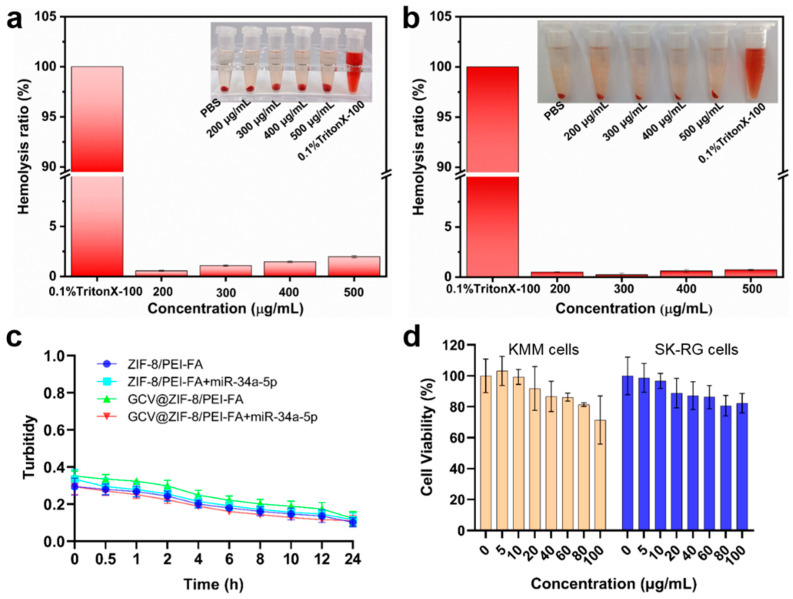
Hemolysis rate, stability, and empty carrier toxicity. (**a**,**b**) Hemolysis rates of ZIF-8/PEI-FA and GCV@ZIF-8/PEI-FA (*n* = 3, mean ± SD), (**c**) turbidity changes in ZIF-8/PEI-FA, GCV@ZIF-8/PEI-FA, ZIF-8/PEI-FA+miR-34a-5p, and GCV@ZIF-8/PEI-FA+miR-34a-5p in 50% FBS (*n* = 3, mean ± SD), and (**d**) cell survival rate of KMM and SK-RG cells (*n* = 5, mean ± SD).

The biosafety of nanocarriers in practical applications is very important, so we tested the hemolysis rate (Equation (3)). *PBS* solution was used as a negative group and 0.1% Triton X-100 as a positive group. As shown in Figure 6a,b, when the concentrations of ZIF-8/PEI-FA and GCV@ZIF-8/PEI-FA were 200 μg/mL, 300 μg/mL, 400 μg/mL, and 500 μg/mL, the color of the supernatant after incubation with red blood cells was similar to that of the negative group. No hemolysis occurred, as in the positive control group. *OD* values of the supernatant after incubation with red blood cells were further detected, all of which were within the safe range (<5%), proving that ZIF-8/PEI-FA has good blood compatibility with GCV@ZIF-8/PEI-FA.

Next, the serum stability was tested by measuring the turbidity changes in the carriers and drug-carrying nanocomplexes in the serum. When ZIF-8/PEI-FA, GCV@ZIF-8/PEI-FA, ZIF-8/PEI-FA+miR-34a-5p, and GCV@ZIF-8/PEI-FA+miR-34a-5p were incubated with 50% FBS, the absorbance (turbidity) changed only slightly within 24 h (Figure 6c). This shows that these nanocarriers have excellent serum stability.

To further detect the biosafety of the nanocarriers, the MTT method was used to detect the cell survival rate of the empty carrier ZIF-8/PEI-FA after incubation with KMM and SK-RG cells. Figure 6d shows that the concentration of ZIF-8/PEI-FA is in the range of 80 μg/mL and is less toxic to both types of cells. In general, all the results show that the nanocarriers prepared in this study have good biocompatibility.

### 2.7. GCV@ZIF-8/PEI-FA Toxicity Analysis of KMM and SK-RG Cells

**Figure 7:**
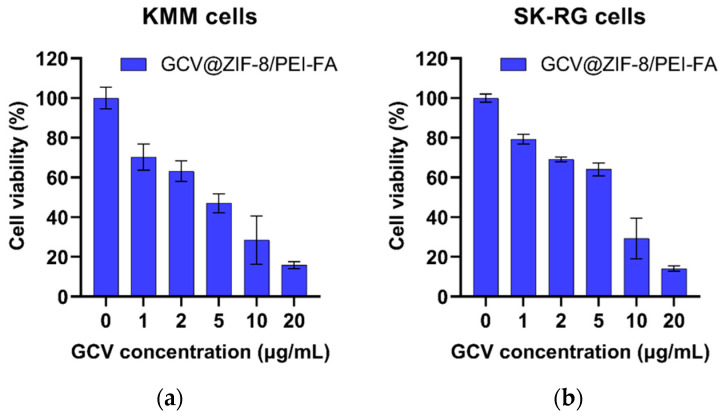
Cytotoxicity. (**a**) Cell survival rate after 48 h incubation of GCV@ZIF-8/PEI-FA with KMM cells and (**b**) cell survival rate after 48 h incubation of GCV@ZIF-8/PEI-FA with SK-RG cells (*n* = 5, mean ± SD).

We further investigated the toxicity of GCV@ZIF-8/PEI-FA to KMM and SK-RG cells. As can be seen from Figure 7a,b, with the increase in the *GCV* concentration, the toxicity of GCV@ZIF-8/PEI-FA to these two kinds of cells increased, showing an obvious drug concentration dependence. The IC_50_ for KMM cells was 3.51 μg/mL, and for SK-RG cells, it was 5.19 μg/mL. This also suggests that when *GCV* is loaded onto the nanocarrier, the amount of *GCV* can produce significant cytotoxicity in a very low concentration range. Therefore, the use of a nanocarrier to load *GCV* is more conducive to the intracellular delivery of GCV.

### 2.8. miR-34a-5p Delivery

**Figure 8:**
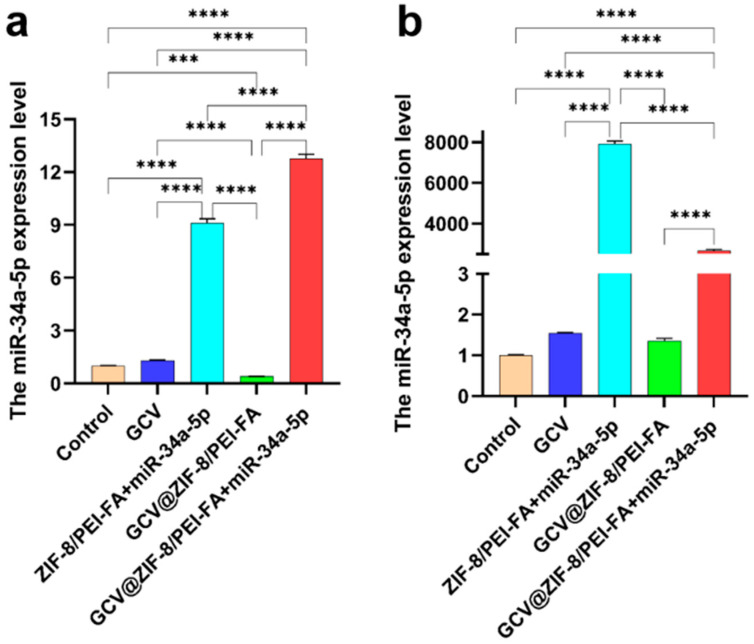
Levels of miR-34a-5p: (**a**,**b**) intracellular miR-34a-5p levels after a treatment of KMM and SK-RG cells with drug-carrying nanocomplexes (*n* = 3, mean ± SD, *** *p* < 0.001, **** *p* < 0.0001).

In order to detect whether the vector could deliver miR-34a-5p into cells and release it, we detected the miR-34a-5p level in the KMM and SK-RG cells via RT-PCR. Compared with the control, GCV, and GCV@ZIF-8/PEI-FA groups, the miR-34a-5p levels of the KMM and SK-RG cells in the ZIF-8/PEI-FA+miR-34a-5p group and the GCV@ZIF-8/PEI-FA+miR-34a-5p group significantly increased (Figure 8a,b). This indicates that the nanocarrier ZIF-8/PEI-FA does indeed have the ability to adsorb and deliver miR-34a-5p into cells. Even if ZIF-8/PEI-FA is already loaded with the drug GCV, it does not affect its ability to deliver miR-34a-5p. It also shows that the nanocomplex can release miR-34a-5p using the “proton sponge effect” of PEI after entering the cell. At the same time, the presence of the small-molecule drug *GCV* does not interfere with miR-34a-5p. 

### 2.9. Cellular Uptake

**Figure 9:**
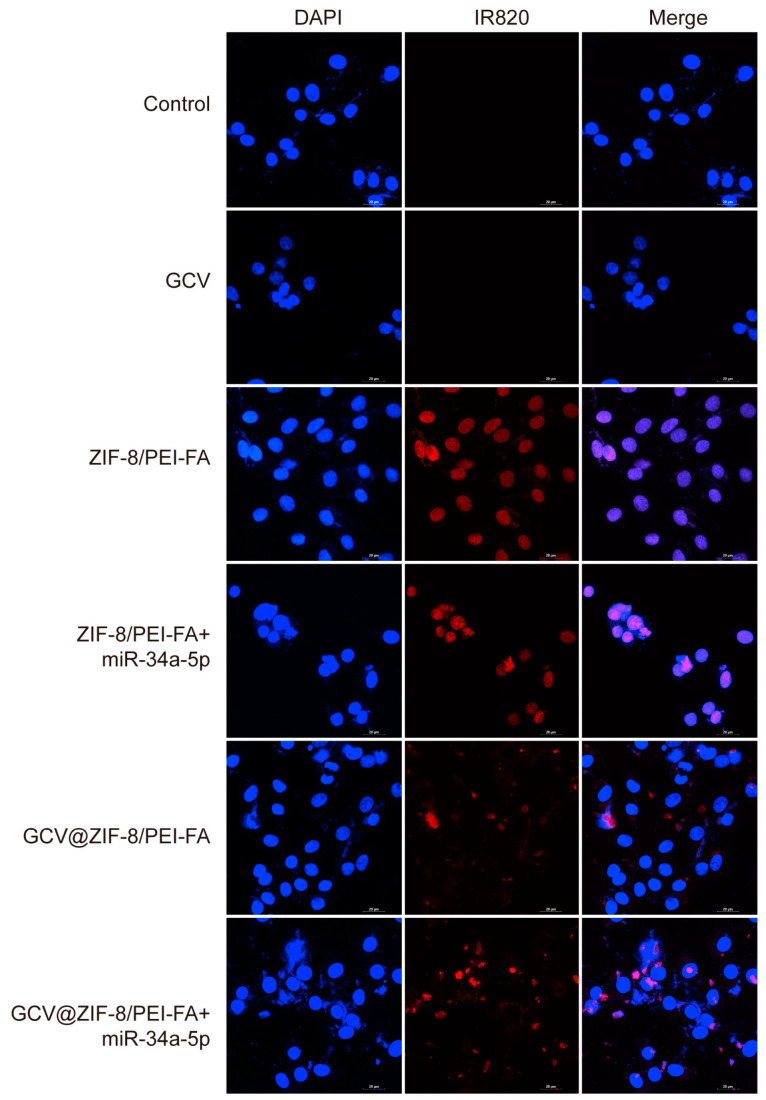
Cellular uptake of drug-carrying nanocomplexes. CLSM image of KMM cells treated with drug-carrying nanocomplexes for 4 h (scale bar: 20 μm).

FA receptors overexpressed on tumor cell membranes can bind specifically to FA molecules modified on drug-carrying nanocomplexes to target tumor cells through FA receptor-mediated endocytosis. To explore the uptake ability, we loaded the red fluorescent dye IR820 into ZIF-8 to prepare ZIF-8/PEI-FA with red fluorescence. KMM cells were treated with ZIF-8/PEI-FA+miR-34a-5p, GCV@ZIF-8/PEI-FA, and GCV@ZIF-8/PEI-FA+miR-34a-5p, and then observed using CLSM (Figure 9). In the KMM cells, due to the presence of PEI-FA in the sample group, this group showed significantly enhanced fluorescence intensity compared with GCV, indicating that the PEI-FA coating layer can increase the carrier’s specific uptake by cells, which is conducive to the intracellular aggregation of *GCV* and miR-34a-5p and improves the utilization rate of the drugs.

### 2.10. Cell Proliferation Effect

**Figure 10:**
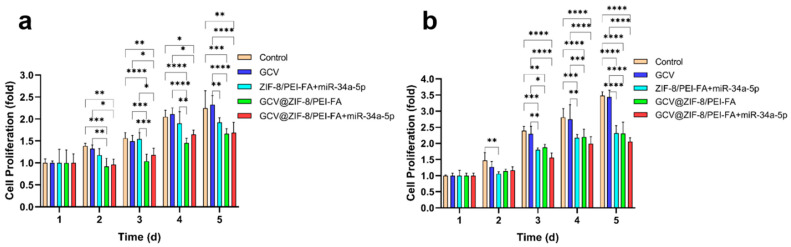
Cell proliferation effects of (**a**) drug-carrying nanocomplexes on the proliferation of KMM cells and (**b**) SK-RG cells (*n* = 5, mean ± SD, * *p* < 0.05, ** *p* < 0.01, *** *p* < 0.001, **** *p* < 0.0001).

The effects of GCV, ZIF-8/PEI-FA+miR-34a-5p, GCV@ZIF-8/PEI-FA, and GCV@ZIF-8/PEI-FA+miR-34a-5p on the proliferation of KMM and SK-RG cells were detected using an MTT assay. As shown in Figure 10a, on day 2, ZIF-8/PEI-FA+miR-34a-5p, GCV@ZIF-8/PEI-FA, and GCV@ZIF-8/PEI-FA+miR-34a-5p inhibited the proliferation of KMM cells. On day 5, the proliferation inhibition rates of ZIF-8/PEI-FA+miR-34a-5p, GCV@ZIF-8/PEI-FA, and GCV@ZIF-8/PEI-FA+miR-34a-5p were 14.46%, 31.93%, and 24.68%, respectively. As shown in Figure 10b, on day 3, ZIF-8/PEI-FA+miR-34a-5p, GCV@ZIF-8/PEI-FA, and GCV@ZIF-8/PEI-FA+miR-34a-5p could inhibit the proliferation of SK-RG cells. On day 5, the inhibition rates of ZIF-8/PEI-FA+miR-34a-5p, GCV@ZIF-8/PEI-FA, and GCV@ZIF-8/PEI-FA+miR-34a-5p were 33.4%, 33.71%, and 40.96%, respectively. These results indicate that the co-loaded dual-drug nanocomplex has the ability to inhibit the proliferation, and the inhibitory effect is better than that of a single drug; thus, the combination of two drugs can play a better role in inhibiting cell proliferation.

### 2.11. Cell Migration and Wound Healing Effect

**Figure 11:**
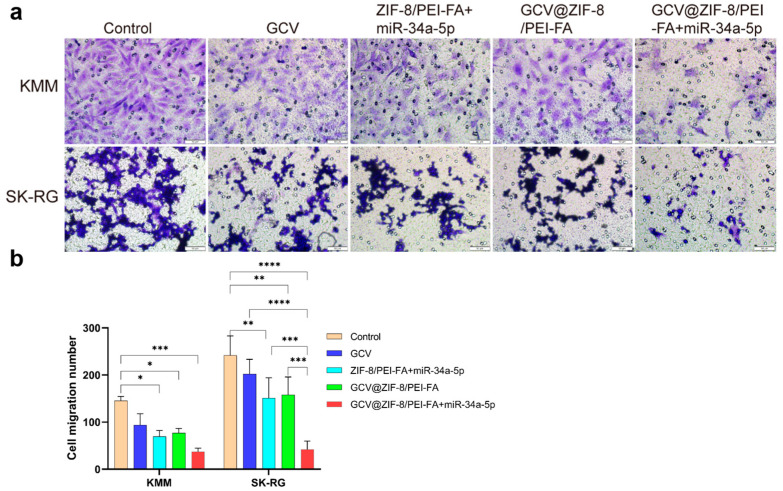
Cell migration. (**a**) Microscopic migration of KMM and SK-RG cells treated with drug-carrying nanocomplexes for 36 h (scale: 50 μm) and (**b**) quantitative cell migration (*n* = 3, mean ± SD, * *p* < 0.05, ** *p* < 0.01, *** *p* < 0.001, **** *p* < 0.0001).

The inhibition of the cell migration ability is one of the methods used to treat tumors. As shown in Figure 11a,b, although *GCV* exerts certain inhibitory effects on KMM and SK-RG cell migration, the effect is not very good. After treatment with ZIF-8/PEI-FA+miR-34a-5p, GCV@ZIF-8/PEI-FA, and GCV@ZIF-8/PEI-FA+miR-34a-5p for 36 h, the migration ability was significantly reduced compared with the control group, showing an obvious inhibitory effect. Moreover, GCV@ZIF-8/PEI-FA+miR-34a-5p showed a more obvious inhibition of cell migration than the other groups, demonstrating that the co-loaded dual-drug nanocomplex has the ability to inhibit cell migration to a considerable extent.

The wound healing assay is another method used to determine the ability of drug-carrying nanocomplexes in inhibiting KMM cell migration. As shown in Figure S2, scratches in the control group gradually narrowed over time. The wound healing rates of the GCV, ZIF-8/PEI-FA+miR-34a-5p, and GCV@ZIF-8/PEI-FA groups were slower than that of the control group, and the fusion rate of the scratches in GCV@ZIF-8/PEI-FA+miR-34a-5p group was slower than those of the other groups over time. Therefore, this further indicates that GCV@ZIF-8/PEI-FA+miR-34a-5p has a better inhibitory effect on cell migration.

### 2.12. KSHV Genes Expression Effect

**Figure 12:**
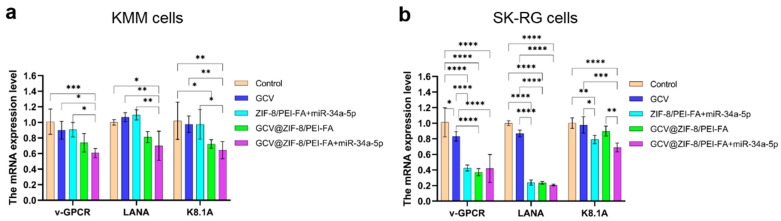
Effects of drug-carrying nanocomplexes on the expression levels of KSHV genes in (**a**) KMM cells and (**b**) SK-RG cells (*n* = 3, mean ± SD, * *p* < 0.05, ** *p* < 0.01, *** *p* < 0.001, **** *p* < 0.0001).

In KSHV-infected cells, the infection includes a latent state and a lytic state. In the latent state, the virus expresses only limited proteins to avoid the host immune response and protect the virus. The lytic viral genome evolves into linear DNA, which facilitates massive viral replication. Therefore, we further detected the expression levels of the KSHV-latent gene LANA and the lytic genes v-GPCR and K8.1A using RT-PCR and verified whether GCV@ZIF-8/PEI-FA+miR-34a-5p can effectively inhibit the expression level of KSHV genes. As shown in Figure 12a,b, GCV@ZIF-8/PEI-FA+miR-34a-5p can effectively inhibit the expression level of the KSHV-latent gene LANA and the lytic genes v-GPCR and K8.1A in KMM and SK-RG cells, and the effect of the combination of the two drugs against KSHV is better than that of a single drug.

## 3. Materials and Methods

### 3.1. Materials

Folic acid (FA), N-N’-dicyclohexyl carbodiimide (DCC), N-hydroxysuccinimide (NHS), branched-chain polyethylenimide (PEI, MW = 1800), dimethyl sulfoxide (DMSO), zinc nitrate hexahydrate (Zn(NO_3_)_2_·6H_2_O), and 2-methylimidazole (2-MIM) were purchased from Aladdin (Shanghai, China). Ganciclovir (GCV) was purchased from Adamas (Shanghai, China), and miR-34a-5p was purchased from the Gima Company (Shanghai, China).

### 3.2. Preparation of ZIF-8/PEI-FA and GCV@ZIF-8/PEI-FA

#### 3.2.1. Preparation of PEI-FA

FA (273 mg) and DCC (125 mg) were dissolved in 15 mL of DMSO, stirred in an oil bath at 60 °C under a N_2_ atmosphere for 15 min away from light, and then, NHS (70.5 mg) was added for 2 h. Then, PEI (1.090 mg) in DMSO (10 mL) solution was slowly added to the mixture and continuously stirred for 24 h. The solution was removed, placed into a dialysis bag (MWCO 2000 Da), and dialyzed with ultra-pure water for 5 days before being freeze-dried to obtain the PEI-FA product.

#### 3.2.2. Preparation of ZIF-8/PEI-FA

(1) (Zn(NO_3_)_2_·6H_2_O) (240 mg) was added to methanol (10 mL) and stirred at room temperature to form a clarified solution. Then, 2-MIM (480 mg) was dissolved in methanol (10 mL) and added to the mixture. After stirring for 12 h, the mixture was centrifuged (8000 rpm, 10 min) with anhydrous ethanol 3 times; the ZIF-8 product was obtained after drying at 40 °C for 12 h in a vacuum drying oven.
(2) The ZIF-8 and PEI-FA were added to *PBS* (at a dosage ratio of 1:2), ultrasound-dissolved, and stirred at room temperature for 24 h. Then, the solution was centrifuged (13,000 rpm, 10 min) 3 times with deionized water, and the obtained ZIF-8/PEI-FA product was freeze-dried.

#### 3.2.3. Preparation of GCV@ZIF-8/PEI-FA 

GCV (100 mg) was dissolved in DMSO (2 mL) and then Zn(NO_3_)_2_·6H_2_O (240 mg) was added to methanol (10 mL) and stirred at room temperature to form a clarified solution. *GCV* (400 μL) was added, and then, 2-MIM was added as described in step (1) in Section 3.2.2 to obtain GCV@ZIF-8.

The GCV@ZIF-8 and PEI-FA were added to the *PBS* (at a dosage ratio of 1:2), ultrasound-dissolved, and stirred at room temperature for 24 h. Then, the solution was centrifuged (13,000 rpm, 10 min) 3 times with deionized water, and the obtained GCV@ZIF-8/PEI-FA product was freeze-dried.

### 3.3. Preparation of ZIF-8/PEI-FA+miR-34a-5p and GCV@ZIF-8/PEI-FA+miR-34a-5p

ZIF-8/PEI-FA and GCV@ZIF-8/PEI-FA were dissolved into *PBS* at a concentration of 1 mg/mL, and then miR-34a-5p (1 μg) was added and swirled for 30 s. After standing for 30 min, ZIF-8/PEI-FA+miR-34a-5p and GCV@ZIF-8/PEI-FA+miR-34a-5p were obtained.

### 3.4. Characterization of Drug-Carrying Nanocomplexes

The product was characterized using ^1^H NMR (AVANCE III HD, Switzerland) with deuterium oxide (D_2_O, 4.70 ppm) as the test solvent. FTIR (VERTEX 70, Bruker, Billerica, MA, USA) and UV-8000S (Yuanxi, Shanghai, China) were used to determine the chemical structure. XRD (D8 Advance, Bruker AXS, Germany) was used to test the crystal structure of the material under the detection condition of Cu Kα rays with the inspection 2θ range of 5 to 90°. TEM (HT7700, Hitachi, Tokyo, Japan) was used to observe the morphology of the sample. The particle size and Zeta potential of the materials were measured using Nanoplus-3.

### 3.5. Drug Loading Rate and In Vitro Release of GCV

#### 3.5.1. Calculation of Drug Loading Rate

GCV@ZIF-8/PEI-FA (1 mg) was dissolved in *PBS* solution (pH = 1.0). Its structure was decomposed via ultrasound, and the *GCV* was completely released. Then, the absorbance value (*OD* 251 nm) was detected, and the *OD* value was substituted into the standard curve established in advance (Figure S1) to calculate the mass of the *GCV* (*n* = 3). The drug loading rate (LD) of the *GCV* was calculated as follows:

(1) $$LD(\%)=\frac{M_{GCV}}{M_{PEI-FA}}\times 100$$

where *M_GCV_* represents the mass of the *GCV* in the GCV@ZIF-8/PEI-FA, and *M_PEI−FA_* represents the mass of the GCV@ZIF-8/PEI-FA.

#### 3.5.2. In Vitro *GCV* Release Experiment

Briefly, 10 mg of GCV@ZIF-8/PEI-FA was dispersed in *PBS* (pH = 7.4 and pH = 5.0, respectively), and placed into a dialysis bag (MW = 300 Da). Then, the dialysis bag was immersed in a centrifuge tube containing 23 mL of two different pH levels of *PBS* solution, and then placed on a shaking table at 37 °C and shaken at 100 rpm. Then, 4 mL of supernatant was absorbed at different time points (total 50 h), and the *OD* value of the supernatant was detected at 251 nm and calculated using a standard curve. The cumulative *GCV* release percentage (E_r_) was calculated according to the following formula:

(2) $$Er(\%)=\frac{V_{e}\sum_{1}^{n-1}c_{i}+V_{0}c_{n}}{M_{GCV}}\times 100$$

where *M_GCV_* represents the mass of the *GCV* in the GCV@ZIF-8/PEI-FA, *V_e_* represents the volume of each sample, *c_i_* represents the concentration of the *GCV* in the i sample, *V*_0_ represents the volume of the *PBS*, and *c_n_* represents the concentration of the *GCV* in the n sample.

### 3.6. Gel Retardation Assay

The miR-34a-5p binding ability with ZIF-8/PEI-FA and GCV@ZIF-8/PEI-FA was detected through a gel retardation assay. Firstly, ZIF-8/PEI-FA, GCV@ZIF-8/PEI-FA were vortexed with miR-34a-5p and then incubated for 30 min at room temperature. Secondly, gel electrophoresis was conducted using a TAE buffer at 110 V for 20 min in a sub-cell system (Bio-Rad Laboratories, Hercules, CA, USA). miR-34a-5p bands were visualized using a UV lamp with a GelDoc system (Synoptics Ltd., Cambridge, UK).

### 3.7. Protection Experiment of miR-34a-5p

Free miR-34a-5p, ZIF-8/PEI-FA+miR-34a-5p, and GCV@ZIF-8/PEI-FA+miR-34a-5p were mixed with 50% FBS or RNase A solution for 1 h before the gel retardation assay (described as Section 3.6).

### 3.8. In Vitro Hemolysis Test

A 5% red blood cell suspension was prepared in advance. ZIF-8/PEI-FA and GCV@ZIF-8/PEI-FA with different concentrations (0.4, 0.6, 0.8, 1.0 mg/mL) were prepared in *PBS* (pH 7.0) solution, with 0.1% Triton X-100 used as positive control and *PBS* solution as negative control. The ZIF-8/PEI-FA, GCV@ZIF-8/PEI-FA, 0.1% Triton X-100, and *PBS* solution were added into a centrifuge tube containing a 5% erythrocyte suspension, and then incubated at 100 rpm at 37 °C for 2 h on a shaking table. Following this, centrifugation at 2000 rpm for 10 min was performed for photo recording, and the supernatant (100 μL) of each group was absorbed into a 96-well plate to determine the *OD* value at 540 nm. The sample hemolysis rate was calculated as follows:

(3) $$\eta \;(\%)=\frac{{OD}_{s}-{OD}_{PBS}}{{OD}_{t}-{OD}_{PBS}}\times 100$$

where *OD_s_* represents the *OD* value of the sample group, *OD_*PBS*_* represents the *PBS* group, and *OD_t_* represents the 0.1% Triton X-100 group.

### 3.9. In Vitro Stability Test

ZIF-8/PEI-FA, ZIF-8/PEI-FA+miR-34a-5p, GCV@ZIF-8/PEI-FA, and GCV@ZIF-8/PEI-FA+miR-34a-5p were dissolved in 50% FBS (1 mg/mL), and then 100 μL of each sample was placed into a 96-well plate and incubated at 37 °C. The *OD* value of the measured solution at 600 nm was taken at different time points. The change in the *OD* value can be regarded as a change in the turbidity of the carrier and drug-carrying nanocarrier.

### 3.10. Cell Culture and Toxicity Test

The SK-RG cells were KSHV-infected SH-SY5Y cells, cultured in complete medium containing 6 μg/mL of puromycin (Gibco) at 37 °C in a 5% CO_2_ incubator. The KMM cells were KSHV-transformed rat primary mesenchymal precursor cells, a gift from Hangzhou Normal University, which were cultured in complete medium containing 150 μg/mL of hygromycin (Solarbio) at 37 °C on a 5% CO_2_ incubator. The toxicities of the ZIF-8/PEI-FA and GCV@ZIF-8/PEI-FA on SK-RG and KMM cells were detected using an MTT assay. SK-RG and KMM cells were placed into 96-well plates at a density of 5000 cells/well and cultured overnight. Different concentrations of ZIF-8/PEI-FA and GCV@ZIF-8/PEI-FA (concentration of GCV) were added, and after culturing for 48 h, 10 μL of the MTT solution was added to each well. After 4 h at 37 °C in the dark, the supernatant was removed and 100 µL of DMSO was added to detect the *OD* value at 490 nm.

### 3.11. Cell Uptake Assay

To observe the cellular uptake, we loaded the new indocyanine green (IR820) into drug-carrying nanocarriers to prepare IR820@ZIF-8/PEI-FA, IR820@ZIF-8/PEI-FA+miR-34a-5p, GCV@IR820@ZIF-8/PEI-FA, and GCV@IR820@ZIF-8/PEI-FA+miR-34a-5p. KMM cells were placed in 24-well plates at a density of 5000 cells/well (a sterile cover glass was placed over each well) and cultured overnight. IR820@ZIF-8/PEI-FA, IR820@ZIF-8/PEI-FA+miR-34a-5p, GCV@IR820@ZIF-8/PEI-FA, and GCV@IR820@ZIF-8/PEI-FA+miR-34a-5p were added and cultured for 4 h, the supernatant was removed, the cells were washed using *PBS*, and 4% paraformaldehyde was then added to each well and fixed at room temperature for 25 min. After fixation, DAPI was added to stain the cells at room temperature for 10 min. Finally, the cover glass was removed for preparation and confocal CLSM was used for observation. Because SK-RG cells have PAN promoters that react to the lytic state and EF-1a promoters that react to the latent state of the virus [38], PAN can express RFP when the virus is lysed, and EF-1a can express GFP, so the cell uptake fluorescence observation was not carried out.

### 3.12. Cell Proliferation Assay

After being co-cultured with miR-34a-5p, ZIF-8/PEI-FA+miR-34a-5p, GCV@ZIF-8/PEI-FA, and GCV@ZIF-8/PEI-FA+miR-34a-5p for 24 h, the KMM and SK-RG cells were seeded into a 96-well plate. Following this, 10 μL/well of the MTT solution was added at different time points, and then the cells were cultured for 4 h at 37 °C with 5% CO_2_. A reader was used to detect the absorbance value at 490 nm.

### 3.13. Transwell and Wound Healing Assay

A total of 1 × 10^4^ KMM and SK-RG cells were seeded into 24-well plate chamber inserts with FBS-free medium after culturing with miR-34a-5p, ZIF-8/PEI-FA+miR-34a-5p, GCV@ZIF-8/PEI-FA, and GCV@ZIF-8/PEI-FA+miR-34a-5p for 4 h, and 800 µL of culture medium containing 10% FBS was added to the lower chamber. After incubation for 36 h, the cells were fixed in 4% polyformaldehyde for 20 min and stained in 0.1% crystal violet for 30 min. Six fields were randomly selected and cells were counted using Image-J (9.0) software. 

KMM cells (1 × 10^6^) were inoculated into a 6-well culture plate after culturing with miR-34a-5p, ZIF-8/PEI-FA+miR-34a-5p, GCV@ZIF-8/PEI-FA, and GCV@ZIF-8/PEI-FA+miR-34a-5p for 4h. When the cells reached a confluency of about 80%, a sterile 1 mL pipette tip was used to scratch the monolayer, and then the cells were photographed using a microscope at 0 h, 12 h, 24 h, and 36 h after scratching at an identical location.

### 3.14. RNA Extraction and Real-Time PCR

The total RNA of SK-RG and KMM cells were extracted using TRIzol reagent (Invitrogen, Waltham, MA, USA). cDNA synthesis was carried out using a RevertAid First Strand cDNA Synthesis kit (Thermo Fisher, Waltham, MA, USA). Real-time PCR was used to detect the cDNA recovered (Qiagen, Venlo, The Netherlands); the primer is shown in Table S1. Then, real-time PCR was used to amplify the target fragment. The reaction conditions were 95 °C for 300 s; 95 °C for 10 s; 57 °C for 30 s; and 72 °C for 35 s, for a total of 40 cycles.

### 3.15. Statistical Analysis

All results are expressed as the mean ± standard deviation. The sample size (n) for the experiments is included in the figure legends. GraphPad Prism 9.0 and Origin 2018 software were used for the statistical analysis, and a multivariate analysis of variance was used to determine the statistical significance between the groups. A *p*-value < 0.05 indicated that the difference was statistically significant.

## 4. Conclusions

A novel FA-targeted nanocarrier was successfully prepared in this study for the simultaneous delivery of the antiviral drug *GCV* and miR-34a-5p. The carrier was combined with miR-34a-5p through electrostatic adsorption to form a co-loaded dual-drug nanocomplex GCV@ZIF-8/PEI-FA+miR-34a-5p. The optimal mass binding ratio was 40:1, and the particle size was 277.4 nm. GCV@ZIF-8/PEI-FA had a drug loading rate of 8.6%. The cumulative release rate of *GCV* was 26.64 ± 0.86% for 50 h in a simulated normal body fluid environment and 72.65 ± 2.59% under slightly acidic conditions, indicating that it has a pH response. In addition, CLSM images showed that the PEI-FA-modified drug-carrying nanocomplexes were readily taken up by cells, demonstrating the targeting effect of FA. A series of cell experiments showed that the co-loaded dual-drug nanocomplex inhibited the proliferation and migration of KSHV-positive cells and reduced the mRNA expression of the KSHV genes, thus demonstrating anti-KSHV activity. This co-loaded dual-drug nanocomplex may provide a promising nanoplatform for the delivery of small-molecule antiviral drugs and nucleic acids and could be an attractive anti-KSHV therapeutic strategy.

## Data Availability

The datasets generated during the current study are available from the corresponding author upon reasonable request.

## Supplementary Materials

The following supporting information can be downloaded at https://www.mdpi.com/article/10.3390/ijms25052932/s1.
