# Supplementary material for: Folate-Targeted Nanocarriers Co-Deliver Ganciclovir and miR-34a-5p for Combined Anti-KSHV Therapy
Source: Int J Mol Sci. 2024 Mar 2;25(5):2932. doi: 10.3390/ijms25052932 (PMC10932146; doi:10.3390/ijms25052932)
Supplement: Supplementary file 1 [file ijms-25-02932-s001.zip › ijms-2834532-supplementary.pdf]

Supplementary Information

Folate-targeted Nanocarriers Co-deliver Ganciclovir and miR-34a-5p for Combined Anti-KSHV Therapy

Fangling Li [1,2,†], Dongdong Cao [1,†], Wenyi Gu [3], Dongmei Li [1,*], Zhiyong Liu [2,*] and Lin Cui [1]

1 Key Laboratory of Xinjiang Endemic and Ethnic Diseases, NHC Key Laboratory of Prevention and Treatment of Central Asia High Incidence Diseases, School of Medicine, Shihezi University, Shihezi 832002, China
2 State Key Laboratory Incubation Base for Green Processing of Chemical Engineering, School of Chemistry and Chemical Engineering, Shihezi University, Shihezi 832003, China
3 Australian Institute for Bioengineering and Nanotechnology (AIBN), University of Queensland (UQ), Corner College and Cooper Roads (Building 75), St Lucia, Brisbane, QLD 4072, Australia
* Correspondence: lidongmei@shzu.edu.cn (D.L.); lzy_tea@shzu.edu.cn (Z.L.)
† These authors contributed equally to this work.

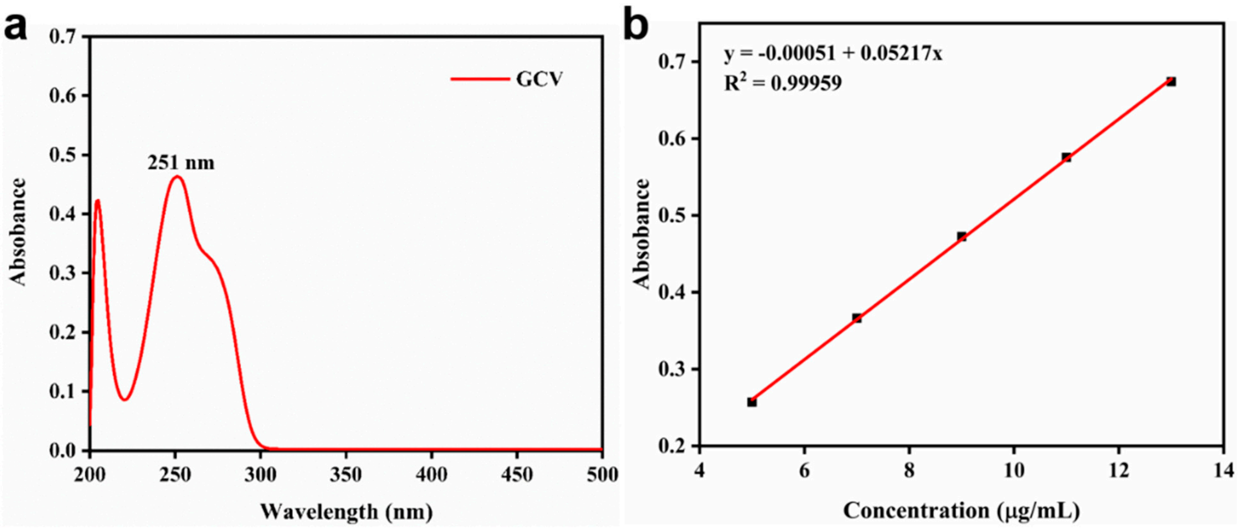

Figure S1 (a) Ultraviolet-visible full-wavelength scanning of GCV, (b) standard curve of GCV and absorbance at different concentrations.

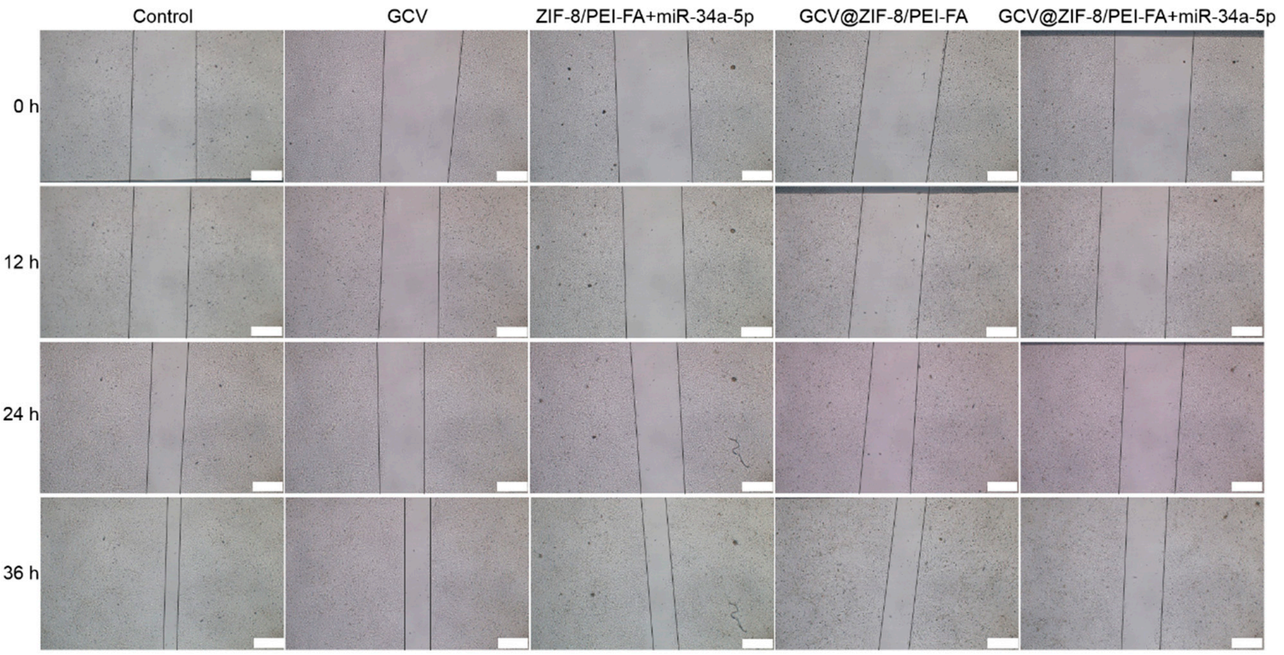

Figure S2 Wound healing assay of KMM cells treated with drug-carrying nanocomplexes for 36 h (scale bar = 500 μm).

Table S1 Primer sequence.

Gene name	Primer sequence (5 '- 3')
K8.1A	F: AAAGCGTCCAGGCCACCACAGA<br>R: GGCAGAAAATGGCACACGGTTAC
v-GPCR	F: GTGCCTTACACGTGGAACGTT<br>R: GGTGACCAATCCATTTCCAAGA
LANA	F:AGCCACCGGTAAAGTAGGAC<br>R:GATGTGACCTTGGCGATGAC
miR-34a-5p	F: CGCGTGGCAGTGTCTTAGCT<br>R: AGTGCAGGGTCCGAGGTATT<br>RT:GTCGTATCCAGTGCAGGGTCCGAGGTATTCGCACTGGATACGACACAACC
U6	F: AGAGAAGATTAGCATGGCCCCTG<br>R: ATCCAGTGCAGGGTCCGAGG<br>RT:GTCGTATCCAGTGCAGGGTCCGAGGTATTCGCACTGGATACGACAAAATA
β-actin	F: CGGAACCGCTCATTGCC<br>R: ACCCACATCGTGCCCATCTA
